# Two-step phase-shifting SPIDER

**DOI:** 10.1038/srep33837

**Published:** 2016-09-26

**Authors:** Shuiqin Zheng, Yi Cai, Xinjian Pan, Xuanke Zeng, Jingzhen Li, Ying Li, Tianlong Zhu, Qinggang Lin, Shixiang Xu

**Affiliations:** 1 Shenzhen Key Lab of Micro-Nano Photonic Information Technology, College of Electronic Science and Technology, Shenzhen University, Shenzhen, Guangdong 518060, People’s Republic of China; 2 SZU-NUS Collaborative Innovation Center for Optoelectronic Science & Technology, Key Laboratory of Optoelectronic Devices and Systems of Ministry of Education and Guangdong Province, Shenzhen University, Guangdong 518060, P. R. China

## Abstract

Comprehensive characterization of ultrafast optical field is critical for ultrashort pulse generation and its application. This paper combines two-step phase-shifting (TSPS) into the spectral phase interferometry for direct electric-field reconstruction (SPIDER) to improve the reconstruction of ultrafast optical-fields. This novel SPIDER can remove experimentally the *dc* portion occurring in traditional SPIDER method by recording two spectral interferograms with π phase-shifting. As a result, the reconstructed results are much less disturbed by the time delay between the test pulse replicas and the temporal widths of the filter window, thus more reliable. What is more, this SPIDER can work efficiently even the time delay is so small or the measured bandwidth is so narrow that strong overlap happens between the *dc* and *ac* portions, which allows it to be able to characterize the test pulses with complicated temporal/spectral structures or narrow bandwidths.

Comprehensive characterization of ultrafast optical fields is critical for ultrashort pulse generation and application, e.g. broadband spectral shaping^1^, quantum coherent control^2^, terahertz pulse generation^3^, super-continuum spectra^4^, strong-field physics^5^, and so on. SPIDER^6^ has been widely used to measure directly both the spectral/temporal phases and the amplitudes of femtosecond pulses, because it can characterize the ultrashort optical pulses from both femtosecond oscillators^7^ and amplifiers^8^ over a wide spectral region from ultraviolet to infrared^9^,^10^. As descripted in ref. 6, the original SPIDER device includes mainly a pulse stretcher, a generator of pulse replicas, a sum-frequency converter and a spectrometer used to record spectral shear interferograms. From the interferograms, one can extract the phase information of test pulses by simple SPIDER algorithm including to use a temporal window to filter out one of the two *ac* components. The two *ac* are separated from the *dc* with an interval of *τ* equal to the time delay between the test pulse replicas. For traditional SPIDER, the delay *τ* and the width of the filter window *τ*_w_ shall be appropriately chosen in order to pick out the *ac* component entirely but clearly. Unfortunately, the choices of both *τ* and *τ*_w_ depend on the spectral or temporal properties of the test pulses, which weakens the reliability of SPIDER measurements.

One of the challenges is to characterize a few-cycle pulse with enormous bandwidth^11^. Besides the need of a Michelson interferometer (MI) with an extra-broadband (up to hundreds of nanometers) 50:50 beam splitter to produce test pulse replicas, the dispersions imposed from the MI on the pulse replicas are also required to be rigorously controlled. Some reported efforts include to modify MI with balanced dispersion between its two arms^7^ or to use a kind of design, called ZAP-SPIDER^12^ without need of MI. For a few-cycle pulse, it is also essential to calibrate and maintain the delay *τ* between the pulse replicas from the generator within interferometric precision, which was realized by using a 2-dimensional spectral shearing interferometry^13^ through scanning the time delay between the largely chirped pulse pair. SEA-SPIDER^14^,^15^ can avoid the rigorous calibration for *τ* with a replacement of a spatial carrier phase calibration. However, the most notable is that a few-cycle pulse usually possesses complicated spectral thus temporal structure, which needs well resolved spectral interference fringes, or small time delay *τ* of the pulse replicas. Unfortunately, small *τ* may lead to the overlapping between the temporal *dc* and *ac* portions from the Fourier transform of the recorded spectral interferogram, which may prevent the SPIDER from working correctly.

Another challenge for SPIDER is to face a test pulse with narrow bandwidth. Narrow bandwidth means large pulse duration, so large *τ* is required to separate clearly the *ac* components from the *dc*, which means the contradiction may occur between *τ* and sampling accuracy of the interferogram similar to the characterization of a few-cycle pulse with enormous bandwidth. For a given spectrometer, narrow bandwidth also implies few sampling points within the effective spectral region of test pulses. Especially, in SPIDER algorithm, the phase concatenation uses only a subset of the available data or the average of all sets to recover spectral phases^6^, which aggravates the insufficiency of the effective sampling points, thereby degrades further the reconstruction quality of the test pulses.

In facing of the two challenges, it is key to avoid the contradiction between *τ* and sampling accuracy of the interferogram. SEA-SPIDER can break the dilemma by using spatial interferogram instead of spectral interferogram. However, this solution simultaneously induces spatial *dc* and *ac* portions from the spatial interferogram, so SEA-SPIDER may be easily effected by spatial diffraction or scattering occurring on the propagation of the test pulses. Meanwhile, SEA-SPIDER needs a 2-dimentional imaging spectrometer, which makes it be much more expensive.

In this paper, we present a novel method to improve the optical-field reconstruction of ultrashort light pulses by combining TSPS^16^ into a SPIDER device, called TSPS-SPIDER. Our results show TSPS-SPIDER can remove experimentally the *dc* portions from the Fourier-transforms of the recorded interferograms, so it works without need of the temporal window and be much insensitive to the time delay *τ*, thus be more reliable. The removal of the *dc* can also avoid the contradiction between *τ* and sampling accuracy of the interferograms, which allows TSPS-SPIDER to be able to characterize the test pulses with complicated temporal/spectral structures or narrow bandwidth. Fortunately, TSPS-SPIDER just needs mainly a traditional SPIDER device, thereby is simple to be implemented.

## Results and Discussion

For a traditional SPIDER, the recorded spectral interferogram *D*(ω_c_) stems from the interference between the sum-frequent pulse pair with a spectral shear Ω and a time interval *τ*, and can be expressed as


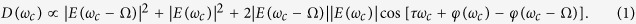


Here *ω*_c_ and *φ*(*ω*_c_) stand for the angular frequency and spectral phase of the optical field *E*(*ω*_c_), respectively. In our setup (see its detail in Method section), an achromatic quarter-wave plate (QW) is inserted in one arm of a MI. Firstly, the QW is aligned so that its incident light can propagate along its fast or slow axis to avoid change the light polarization, and the recorded spectral interferogram (*I*1) can be descripted by equation (1). Then, the QW is rotated by 90° in order that the test light reflected back to the QW by a mirror (MI) will be added another π phase-shifting, so the corresponding spectral interferogram to be recorded (*I*2) becomes


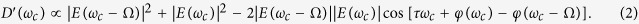


Accordingly,


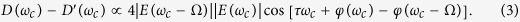


It is well known that after applying Fourier transform to *D*(*ω*_*c*_) or *D*′(*ω*_*c*_) with respect to frequency, the amplitude of corresponding function *D*(*t*) or *D*′(*t*) in time domain includes three parts: One is centered at *t* = 0 (*dc* portion from |*E*(*ω*_*c*_ − Ω)|^2^ + |*E*(*ω*_*c*_)|^2^) while the other two (*ac* components from |*E*(*ω*_*c*_ − Ω) *E*(*ω*_*c*_)|cos[*τω*_*c*_ + *φ*(*ω*_*c*_) − φ(*ω*_*c*_ − Ω)]) are centered at *t* = ±*τ*, respectively. In traditional SPIDER algorithms, proper *τ* is required in order that all the three components are well separated temporally, meanwhile, the recorded spectral interferogram has enough sampling points per fringe in order to suppress the effects of uncorrelated noises which diminish with the square root of the sampling point number^1^. Interestingly, when subtracting *D*′(*t*) from *D*(*t*), we will see the *dc* portion disappears in *D*(*t*) − *D*′(*t*), so theoretically we can remove the effect from the *dc*, thus have great flexibility to choose *τ*. Furthermore, we can get any one of the *ac* components by simply using the data from 0 to +∞ or 0 to −∞ without need of a filter window which is used in traditional SPIDER algorithms.

**Figure 1:**
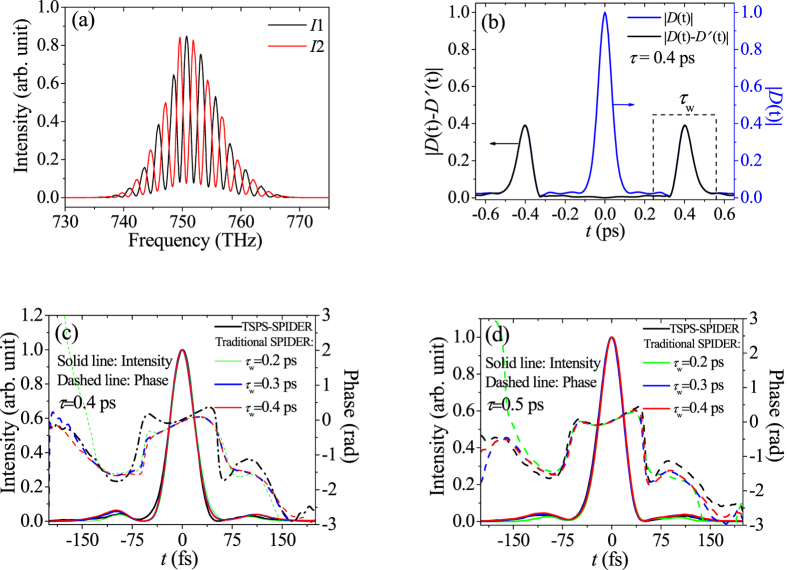
The two measured spectral interferograms *I*1 (black line) and *I*2 (red line) with π phase-difference (**a**); |*D*(*t*)| (blue line) and |*D*(*t*) − *D*′(*t*)| (black line) (**b**); the reconstructed temporal intensity and phase profiles with different filter windows: *τ*_*w*_ = 0.2, 0.3 and 0.4 ps when *τ* = 0.4 ps (**c**); and the reconstructed results under the same conditions of (**c**) except *τ* = 0.5 ps (**d**).

Figure 1(a) shows two recorded spectral interferograms (*I*1 and *I*2). During recording *I*1 and *I*2, the only difference is the test sub-pulse reflected from M1 (see Section Method) has π phase-shifting by rotating the QW with 90°. As predicted, Fig. 1(a) reflects clearly the π phase-difference between the two interferograms. Here, the test pulse is an 800 nm femtosecond pulse train with a bandwidth of 26 nm and the delay *τ* between the pulse replicas is chosen to be 0.4 ps. In Fig. 1(b), the blue line is |*D*(*t*)|, while the black line is |*D*(*t*) − *D*′(*t*)| according to equations (1, 2, 3). The dotted frame illustrates for the filter window needed in traditional SPIDER. We can see the *ac* components of the black and blue lines are well coincided. However, compared with the blue line, the black line keeps the values very close to zero in the region from −0.32 to 0.32 ps, that is to say, the *dc* component in blue line is well removed experimentally. This phenomenon, in turn, is a good evidence of the π phase-difference between the two interferograms in Fig. 1(a). Figure 1(c) presents some results of the electric-field reconstruction by traditional SPIDER with different temporal widths of the filter windows (*τ*_w_ = 0.2, 0.3 and 0.4 ps) and our TSPS-SPIDER (black line). The measured pulse duration (FWHM) is about 42 fs. The inconsistencies are observable among the measured temporal phases (green, blue and red dashed lines) with different values of *τ*_w_ by traditional SPIDER method. Comparatively speaking, the difference is more obvious between the three dashed lines (the green, blue and red lines) and the black dashed line. However, if *τ* increases to 0.5 ps, as shown in Fig. 1(d), the temporal phase lines are well coincided in the temporal region from −0.15 to 0.15 ps for the different filter windows by traditional SPIDER method, and all of them go very closely to that by our TSPS-SPIDER. On these grounds, the inconsistencies among the green, blue and red dashed lines in Fig. 1(c) implies that different width of filter window may introduce different phase errors because the picked *ac* component isn’t entire and clear enough^17^, whereas the difference of the phases by the traditional SPIDER from by the TSPS-SPIDER shall be attributed to the removal of the *dc*. Accordingly, the TSPS-SPIDER can avoid efficiently the effects not only from the filter window width but also from the time delay *τ*.

**Figure 2:**
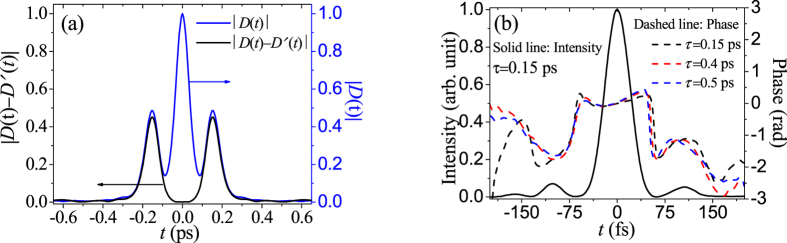
The |*D*(*t*) − *D*′(*t*)| (black line) and *|D*(*t*)*|* (blue line) when *τ* = 0.15 ps (**a**), the reconstructed temporal intensity and phase profiles (black lines) by TSPS-SPIDER (**b**). The temporal phases are also presented with red dashed line when *τ* = 0.4 ps and blue dashed line when *τ* = 0.5 ps for comparison.

As we know, if *τ* is small enough, as shown in Fig. 2(a) (*τ* = 0.15 ps), the *dc* and *ac* components of |*D*(*t*)| will fail to separate entirely (blue line), so traditional SPIDER cannot work accurately, even correctly. Interestingly, the amplitude distribution of |*D*(*t*) − *D*′(*t*)| shows the two *ac* components centered at *t* = ±*τ* are well separated at the absence of the *dc*, so it is still possible to carry out the electrical-field reconstruction of ultrashort light pulses. Figure 2(b) presents the corresponding temporal phase and intensity profiles using TSPS-SPIDER by the black lines: solid line for intensity and dashed line for phase. For convenience of comparison, the phase information of the optical field is also recovered with *τ*  = 0.4 ps (the red dashed line) and 0.5 ps (the blue dashed line) by TSPS-SPIDER, as shown in Fig. 2(b). One can see that the three temporal phases are coincident with each other, especially in the region from −100 to 100 fs, when the value of *τ* ranges from 0.15 ps to 0.5 ps. These results prove the TSPS-SPIDER is still available even *τ* is so small that the *dc* and the *ac* portions of |*D*(*t*)| or |*D*′(*t*)| overlap greatly with each other.

**Figure 3:**
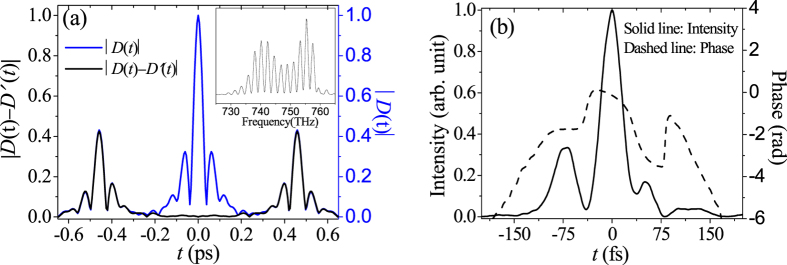
The recorded spectral interferogram *I*1 (inset) and the |*D*(*t*) − *D*′(*t*)| (black line) and |*D*(*t*)| (blue line) when *τ* = 0.45 ps (**a**), and the reconstructed temporal intensity and phase profiles by TSPS-SPIDER (**b**).

Figure 2 predicts that the TSPS-SPIDER has obvious advantage over the traditional SPIDER in characterizing the ultrashort pulses with complicated spectral structures, where the fine spectral amplitude modulation requires the small time delay *τ* between the test pulse replicas. In order to generate the test pulses with complicated spectral structures, a 2.5 mm-thick CaF_2_ crystal is used as Kerr medium. The spectral interferogram is shown in the inset of Fig. 3(a). The complicated spectral structures usually result in complicated temporal structure, so after Fourier-transform, as shown in Fig. 3(a) where *τ* = 0.45 ps, both the *dc* and the *ac* have complicated temporal structures: multi-peaks and long pre/post backgrounds, which leads to the strong overlap with each other. Even so, the black line in Fig. 3(a) shows our TSPS-SPIDER has got a perfect removal of the *dc*, so we are still able to recover the spectral/temporal phases and intensities of the test pulses. Figure 3(b) uses dashed line for the temporal phase and solid line for intensity profile by our TSPS-SPIDER, which agrees with our numerical simulation very well. One can see that the self-phase modulation by using the Kerr medium results in the test pulse with complicated temporal intensity and phase profiles.

**Figure 4:**
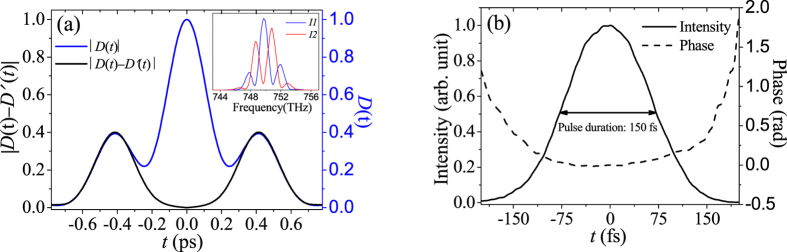
The recorded spectral interferograms *I*1 and *I*2 (inset) and the |*D*(*t*) − *D*′(*t*)| (black line) and |*D*(*t*)| (blue line) when *τ* = 0.40 ps (**a**), and the reconstructed temporal intensity and phase profile by TSPS-SPIDER method (**b**).

It is predictable that our TSPS-SPIDER has stronger ability than traditional SPIDER to measure the test pulse with narrower bandwidth. The narrower bandwidth means larger transform-limited pulse duration, so the traditional SPIDER method needs large time delay *τ*, which may result in the recorded interference fringes to be unresolvable with the available spectrometer, or the significantly increasing effects of uncorrelated noise which diminish as the square root of the number of points per fringe. For example, in Fig. 4(a), we reduce the bandwidth of test pulse down to 7.0 nm by using a bandpass filter, so the corresponding pulse duration shall be not less than 134 fs. When *τ* = 0.40 ps, there are about 51 sampling points per fringe. Unfortunately, the very strong overlap occurs between the *dc* and *ac* portions. In order to avoid the overlap, we estimate that the time delay *τ* of the test pulse replicas shall be as large as 2.5 ps^18^. Of course, in this situation, a grating pulse stretcher shall be used instead of the prism pair, and the sampling point will drop to about 8 per fringe. However, by our TSPS-SPIDER, the two *ac* components centered at *τ* = ±0.40 ps are well separated temporally, so we can reconstruct the temporal intensity and phase information (see Fig. 4(b)) easily without replacement of the pulse stretcher, meanwhile, the sampling point can maintain about 50 per fringe, which is beneficial to suppress the effect of the uncorrelated noise.

## Conclusions

In this paper, we present an improved optical-field reconstruction of ultrashort pulses by TSPS-SPIDER. This method works by recording two spectral interferograms with π phase-shifting. Our results show it can remove experimentally the effect of the *dc* portion occurring in traditional SPIDER method. Compared with the traditional SPIDER, TSPS-SPIDER makes the reconstructed results be much less disturbed by the time delay *τ* between the test pulse replicas and the temporal width of the filter window *τ*_w_, so the measurements become more reliable. What is more, no contribution of the *dc* allows the SPIDER device to work efficiently even the time delay *τ* is so small that strong overlap happens between the *dc* and *ac* portions. Consequently, TSPS-SPIDER has more flexible choice for *τ*, which extends no doubt its ability to characterize the pulses with complicated temporal/spectral structures or narrow bandwidths. We believe this work will not only expand the application fields of SPIDER, but also be very helpful to improve the diagnosis quality of ultrashort pulses for the laboratories possessing a traditional SPIDER device, because our TSPS-SPIDER just needs actually a traditional SPIDER device plus an achromatic quarter-wave plate. Additionally, the subtraction from two interferograms *I*1 and *I*2 recorded by using one receiver at different time is somewhat equivalent to that of a balanced detection where the balanced output is the difference between the two signals recorded by two similar receivers. It is well known that balanced detection is widely used to cancel the intensity noise intrinsic to input pulses and the noises common to both detectors^19^. In our experiments, the pairs of the interferograms have been recorded within an interval of about tens of seconds, so the subtraction can cancel the low frequent (<0.02 Hz) noises both from the input laser intensity and from the receiver. During the measurements for Figs 1, 2, 3 and 4, we find the subtraction of *I*2 from *I*1 reduces the noises by a factor from 0.22 to 0.27 compared with corresponding *I*1 or *I*2, thus suppress partially the measured noises. In principle, TSPS can also be used to work with SEA-SPIDER to remove the spatial *dc* portion experimentally. According to the design as shown in Fig. 1 of the ref. 15, we can record the two spatially encoded interferograms with π phase-shifting by just inserting a broadband half-wave plate to one of the two incident sum-frequency pulses to the used imaging spectrometer.

## Methods

**Figure 5:**
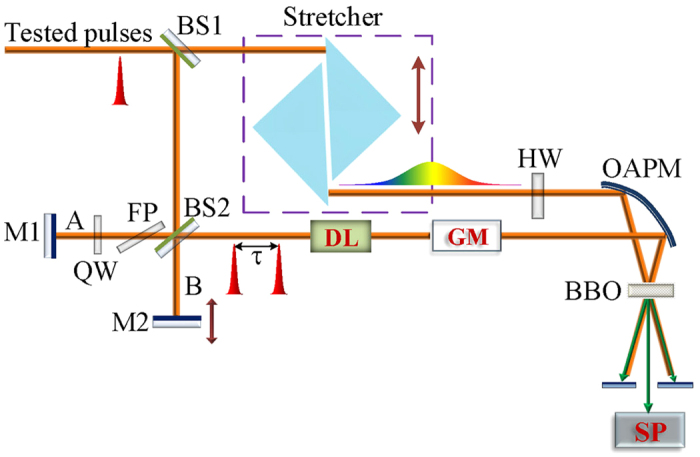
The schematic illustration of our SPIDER setup. BS1, BS2: beam splitters; M1, M2: mirror; DL: temporal delay line; Stretcher: pulse stretcher; HW: achromatic half-wave plate; QW: achromatic quarter-wave plate; OAPM: 90° off-axis parabolic mirror; BBO: 42.3°-cut *β*-BBO crystal for sum-frequency conversion; FP: fused silica plate; SP: fiber spectrometer.

We implement our TSPS-SPIDER by the setup as shown in Fig. 5 which is a typical SPIDER except adding an achromatic quart-wave plate (QW) and a fused silica plate (FP) in one arm (A) of the MI. Here the QW is firstly aligned with its fast axis being parallel to the polarization of the double-passing laser beam, so the beam can get an additional π phase-shifting without change of its polarization by rotating the QW with 90°. The BS2, a 2 mm-thick broadband 50:50 beam splitter made of fused silica, is aligned so that its reflecting surface faces towards arm A. The FP is used to balance the total dispersion of the arm A with that of arm B, so our setup is qualified for few-cycle pulses. Our QW includes a 0.50 mm-thick MgF_2_ plate and a 0.61 mm-thick quartz plate, so we can figure out the required thickness of the FP shall be about 1.405 mm under Brewster angle incidence. With a dispersion-balanced MI, the SPIDER setup can be regarded as a glass plate with the dispersion of one of the two arms followed by the SPIDER setup but with a zero-thick BS2. Our pulse stretcher consists of a pair of 180° folding right-angle prisms (FRAP) made of SF57 glass which are displaced by face to face. This stretcher is very compact, easy to align, but can work with variable dispersion by shifting one of the prism as shown in Fig. 5 thereby changing the displacement between their apexes. By using the stretcher, the 800 nm laser pulses with a bandwidth of 26 nm can be stretched from 2.4 to 15 ps. The achromatic half-wave plate (HW) is used to rotate the polarization of the stretched pulse by 90° in order for the sum-frequency conversions between the chirped pulse and the test pulse replicas via type-II phase-matching. The spectrometer used to record the sum-frequent pulse pair is a fiber spectrometer (HR 4000, Ocean Optics) with a resolution of 0.05 nm.

## Additional Information

**How to cite this article**: Zheng, S. *et al*. Two-step phase-shifting SPIDER. *Sci. Rep.* **6**, 33837; doi: 10.1038/srep33837 (2016).
